# Seroprevalence of COVID-19 in Oran: Cross-Sectional Study

**DOI:** 10.1128/spectrum.00876-23

**Published:** 2023-06-07

**Authors:** Abdessamad Dali-Ali, Dalia Kheira Derkaoui, Mohamed Zina, Asmaa Oukebdane

**Affiliations:** a University of Oran 1: Ahmed Ben Bella, Faculty of Medicine, Oran, Algeria; b Department of Epidemiology and Preventive Medicine at EHUO, Oran, Algeria; c University of Oran 2: Faculty of Sciences, Department of Biology, Oran, Algeria; d Public Establishment of Proximity Care in Boutlellis, Department of Epidemiology and Preventive Medicine, Boutlellis, Oran, Algeria; e Canastel Specialized Hospital, Ophthalmology Department, Oran, Algeria; Keck School of Medicine of the University of Southern California

**Keywords:** cross-sectional study, Algeria, seroprevalence, COVID-19, SARS-CoV-2, Oran

## Abstract

The coronavirus disease 2019 (COVID-19) pandemic caused by severe acute respiratory syndrome coronavirus 2 (SARS-CoV-2) was introduced in Algeria in March 2020. This study aimed to estimate the seroprevalence of SARS-CoV-2 infection in Oran, Algeria, and to identify factors associated with seropositivity. This was a cross-sectional seroprevalence study conducted between 7 and 20 January 2021 across all 26 municipalities in the province of Oran. The study employed a random cluster sampling technique stratified by age and sex to select participants from households, who were then administered a rapid serological test. The overall seroprevalence and specific seroprevalences by municipality were calculated, and the number of COVID-19 cases in Oran was estimated. The correlation between population density and seroprevalence was also examined. Among the participants, 422 (35.6%; 95% confidence interval [CI], 32.9 to 38.4) had a positive serological test for SARS-CoV-2, and eight municipalities had seroprevalence rates above 73%. We found a strong positive correlation between population density and seroprevalence (*r* = 0.795, *P* < 0.001), indicating that areas with higher population density had higher numbers of positive COVID-19 cases. Our study provides evidence of a high seroprevalence of SARS-CoV-2 infection in Oran, Algeria. The estimated number of cases based on seroprevalence is much higher than the number of cases confirmed by PCR. Our findings suggest that a large proportion of the population has been infected with SARS-CoV-2, highlighting the need for continued surveillance and control measures to prevent further spread of the virus.

**IMPORTANCE** This is the first and only seroprevalence study of COVID-19 conducted in the general population in Algeria prior to the national vaccination campaign against COVID-19. The significance of this study lies in its contribution to our understanding of the spread of the virus in the population before the implementation of the vaccination program.

## INTRODUCTION

As of 5 July 2021, more than 183 million confirmed cases of coronavirus disease 2019 (COVID-19) had been reported to the World Health Organization (WHO), with 3,978,581 deaths, and 2,988,941,529 doses of vaccine administered (1). The United States ranked first with over 33,378,423 cases, followed by India (30,585,229 cases) and Brazil (18,742,259 cases) (1).

In Algeria, the first imported case was recorded on 25 February 2020 in the province of Ouargla in an Italian national. However, the first indigenous outbreak was reported in the province of Blida on 1 March 2020, marking the effective start of the epidemic (2). Between March 2020 and March 2021, Algeria experienced two waves of the COVID-19 epidemic caused by the Alpha variant of severe acute respiratory syndrome coronavirus 2 (SARS-CoV-2). The second wave began in October 2020 and continued until March 2021 (1).

As of 28 June 2021, there were 138,840 PCR-confirmed cases of COVID-19 in Algeria, with an incidence rate of 325.92 cases per 100,000 population (2). The central health region had the highest incidence rate (385.09 per 100,000 inhabitants), followed by the western, eastern, and southern health regions with incidence rates of 310.00, 308.58, and 230.59 per 100,000 inhabitants, respectively (2). However, it is the province of Oran that has always ranked first, with a cumulative incidence rate equal to 742.69 per 100,000 inhabitants (2).

The spread of the disease within the population is estimated based on confirmation of infection by PCR in symptomatic cases, which excludes asymptomatic individuals or those with mild symptoms who contribute to the spread of the virus. In fact, some studies suggest that the number of asymptomatic cases exceeds that of symptomatic cases (3, 4), highlighting the importance of conducting seroprevalence surveys based on the detection of anti-SARS-CoV-2 antibodies (5). To do so, the random selection of a representative sample of the general population, as well as the use of an accurate screening test, allows the estimation of the real extent of the epidemic within a given region (6).

In the absence of a seroprevalence survey conducted to date in Algeria and based on a working hypothesis stating a high prevalence of COVID-19, the objective of our study was to determine the seroprevalence of SARS-CoV-2 infection within the population of Oran, as well as the factors associated with a positive seroprevalence.

## RESULTS

### Steps of study participation.

**FIG 1:**
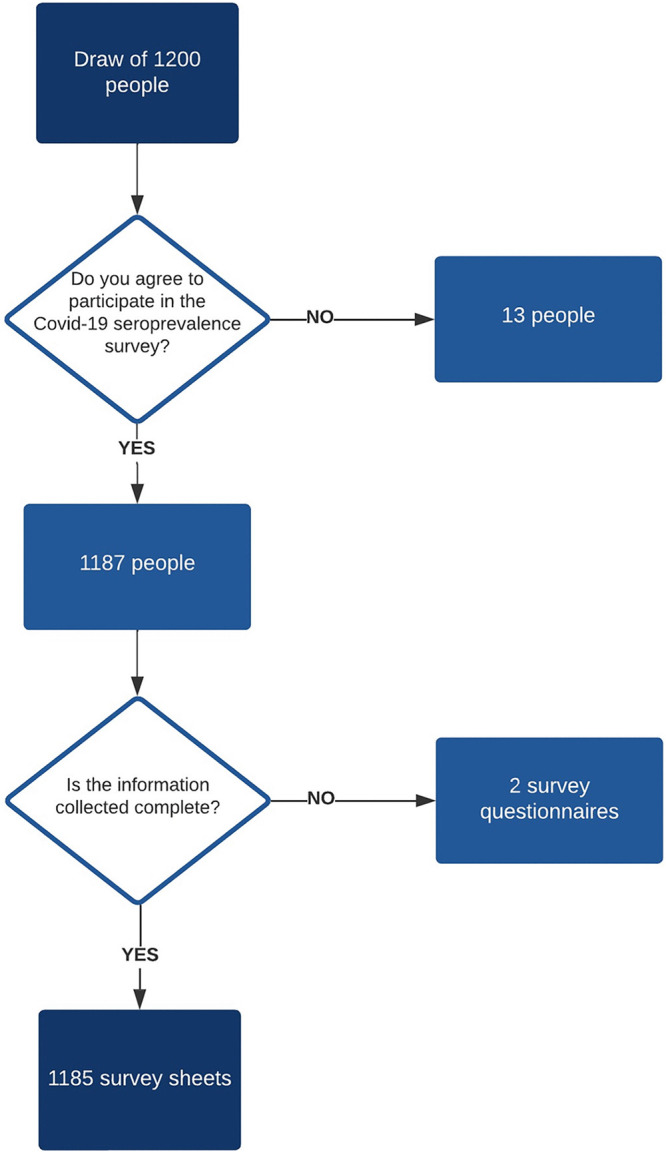
COVID-19 seroprevalence survey: flowchart.

Out of 1,200 subjects randomly selected, 1,187 subjects agreed to participate in the study (13 refusals), resulting in a participation rate of 98.9%. After checking the survey forms, we found that two forms had a significant amount of missing data, leading to their exclusion from the analysis. In total, the survey included 1,185 subjects (Fig. 1).

### Descriptive data.

The study enrolled 1,185 participants with a mean age of 39.3 ± 21.6 years (mean ± standard deviation). The age group with the highest representation was individuals aged 10 to 19 years. Comparison of age means between males (39.6 ± 22.3 years) and females (39.0 ± 20.9 years) showed no statistically significant difference (*P* = 0.62).

The most frequent symptoms were represented by headaches (19.2%), cough (16.0%), and fatigue (15.4%). Anosmia and ageusia accounted for 7.8% and 7.0% respectively (Table 1).

**TABLE 1:** Characteristics of the study population

Variable	No. of participants (*n* = 1,185)	%
Sex		
Female	598	50.5
Male	587	49.5
Age (yrs)		
<10	86	7.3
10–19	217	18.3
20–29	119	10.0
33–39	162	13.7
40–49	189	15.9
50–59	183	15.4
60–69	129	10.9
70–79	65	5.5
≥80	35	3.0
Exposure to SARS-CoV-2		
Yes	197	16.6
No	988	83.4
Type of contact (*n* = 197)		
Family	107	54.3
Professional	40	20.3
Friends	9	4.6
Neighborhood	5	2.5
Other	36	18.3
Presence of symptoms suggestive of COVID-19 in 2020		
Yes	376	31.7
No	809	68.3
Symptoms suggestive of COVID-19 (*n* = 376)		
Headache	227	19.2
Cough	190	16.0
Fatigue	182	15.4
Fever	163	13.8
Shortness of breath	106	8.9
Anosmia	92	7.8
Ageusia	83	7.0
Diarrhea	68	5.7
Nausea/Vomiting	52	4.4
Muscle pain	8	0.7
Chills	5	0.4
Presence of comorbidities		
Yes	251	21.2
No	934	78.8
Types of comorbidities		
Hypertension	154	13.0
Diabetes	99	8.4
Cardiovascular diseases	28	2.4
Allergies	17	1.4
Respiratory diseases	12	1.0
Pregnancy	7	0.6
Anemia	4	0.3
Rheumatic diseases	4	0.3

Of the participants, 197 individuals reported exposure to SARS-CoV-2, with family exposure being the most common (54.3%), followed by occupational exposure (20.3%) (Table 1).

A relatively high prevalence of underlying health conditions was observed in the study sample, with comorbidities present in 251 individuals, accounting for 21.2% of the study population. The most common comorbidities identified in the study population were arterial hypertension (13.8%), diabetes mellitus (8.4%), and cardiovascular diseases (2.4%) (Table 1).

### Seroprevalence results.

According to the data, 8.9% (106 individuals) of the sample reported undergoing a COVID-19 screening or diagnostic test in 2020. Among those who were tested, the most common type of test was serology (5.4%), followed by PCR (3.2%), CT scan (1.7%), and antigen test (0.6%), as shown in Table 2. Of the survey participants who underwent serological testing, 422 tested positive for COVID-19 antibodies. This corresponds to a seroprevalence rate of 35.6% (95% confidence interval [CI], 32.9 to 38.4), indicating that over a third of individuals who underwent serological testing had been infected with COVID-19 at some point prior to the test (Table 2).

**TABLE 2:** Results of the seroprevalence study

Variable	No. of participants (*n* = 1,185)	%
Underwent a screening and/or diagnostic test for COVID-19 in 2020		
Yes	106	8.9
No	1,079	91.1
Types of screening and/or diagnostic tests for COVID-19 in 2020 (*n* = 106)		
Serology	64	5.4
PCR	38	3.2
CT scan	20	1.7
Antigen test	7	0.6
Results of rapid serological test of the prevalence survey (*n* = 1,185)		
Positive	422	35.6
Negative	763	64.4
Detailed antibody results (*n* = 422)		
IgM^+^ IgG^+^	139	11.7
IgM^+^ IgG^−^	40	3.4
IgM^−^ IgG^+^	243	20.5
IgM^−^ IgG^−^	763	64.4

**FIG 2:**
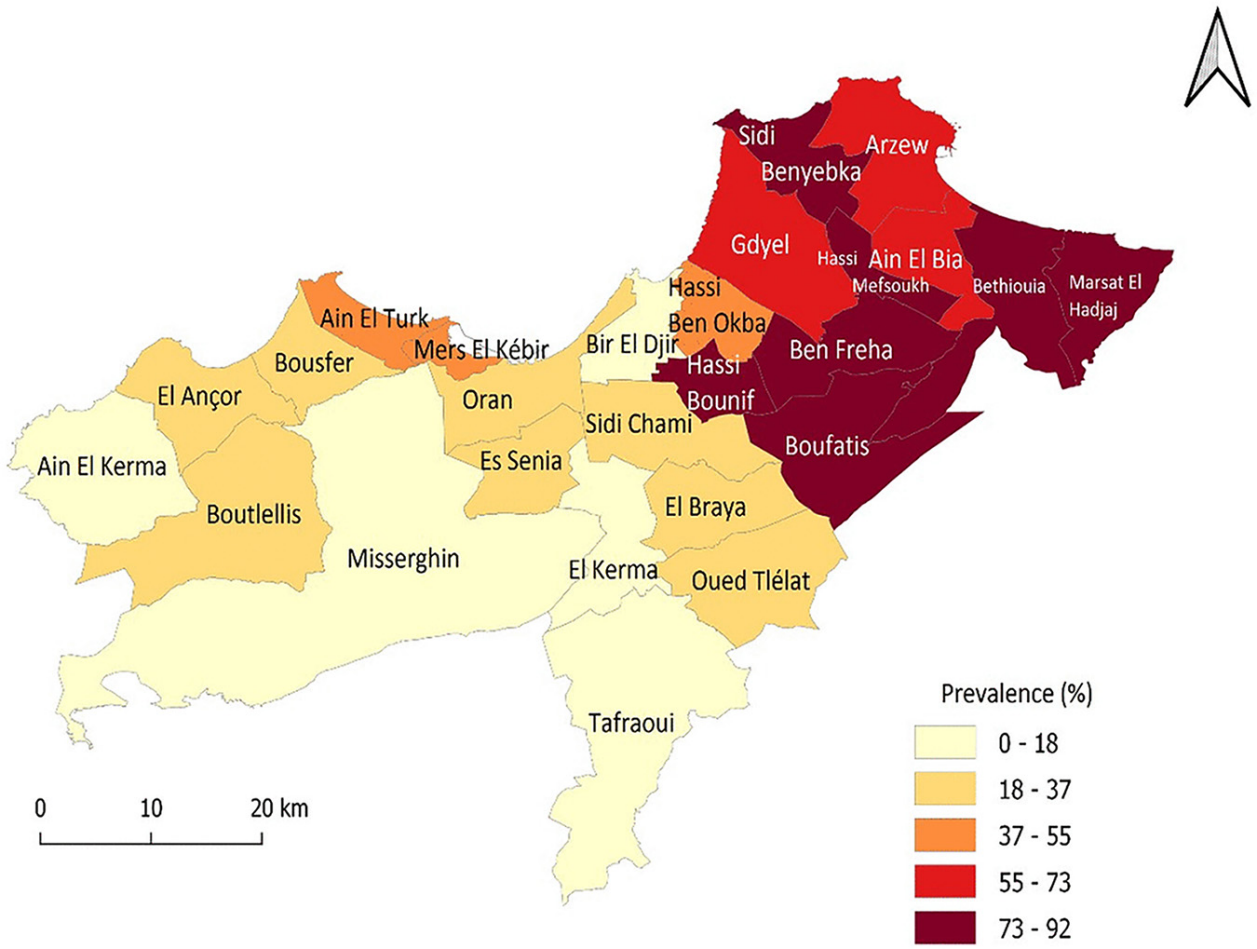
Seroprevalence of COVID-19 in the province of Oran by municipality.

It should be noted that eight communes had seroprevalence rates higher than 73%, namely, Marsat El Hadjaj, Bethioua, Benfreha, Sidi Ben Yabka, Hassi Bounif, Boufatis, Hassi Mefsoukh, and Gdyel (Fig. 2).

However, the study of the correlation between population density and the number of positive COVID-19 rapid tests showed a highly significant relationship between the two variables, with a Spearman’s rank correlation coefficient of 0.795 (*P* < 0.001) (Table 3).

**TABLE 3:** Prevalence of COVID-19 in Oran Province by municipality

Municipality	Area (km^2^)	Population	Population density (inhabitants/km^2^)	Sample size	No. of positive tests	Prevalence (%)	95% CI
Oran	64.00	674,273	10,535.52	308	104	33.8	28.71–39.22
Bir El Djir	32.46	171,883	5,295.22	257	27	10.5	7.32–14.85
Sidi Chahmi	69.50	114,050	1,641.01	118	39	33.1	25.22–41.95
Es Senia	48.51	97,500	2,009.89	77	19	24.7	16.4–35.35
Arzew	71.90	85,658	1,191.35	64	43	67.2	55.0–77,43
Hassi Bounif	31.77	63,581	2,001.29	53	41	77.4	64.47–86.54
Gdyel	93.82	39,129	417.06	30	22	73.3	55.55–85.82
Ain El Turk	39.14	35,687	911.78	29	13	44.8	28.41–62.45
El Kerma	63.55	25,636	403.40	26	0	0	0–12.87
Ain El Bia	36.15	32,611	902.10	24	17	70.8	50.83–85.08
Misserghin	428.28	26,554	62.00	23	4	17.4	6.98–37.14
Benfreha	69.29	23,254	335.60	21	18	85.7	65.37–95.02
Boutlelis	135.97	23,920	175.92	19	5	26.3	11.81–48.79
Bousfer	46.20	18,361	397.42	17	6	35.3	17.31–58.7
Oued Tlelat	84.11	19,384	230.46	15	5	33.3	15,18–58.29
Hassi Mefsoukh	25.67	12,836	500.04	12	9	75.0	46.77–91.11
Mers El Hedjaj	52.29	13,153	251.54	12	11	91.7	64.61–98.51
Mers El Kebir	10.98	17,957	1,635.43	12	6	50.0	25.38–74.62
Hassi Ben Okba	37.47	13,905	371.10	12	6	50.0	25.38–74.62
Bethioua	108.57	18,215	167.77	11	10	90.9	62.27–98.38
Tafraoui	182.00	12,089	66.42	10	1	10.0	1.79–40.41
El Onçor	66.44	11,469	172.62	10	3	30.0	10.78–60.32
Boufatis	99.06	11,872	119.85	8	6	75.0	40.93–92.85
El Braya	57.26	6,292	109.88	6	2	33.3	9.68–70.0
Sidi Benyebkra	51.69	7,825	151.38	6	5	83.3	43.65–96.99
Ain El Kerma	107.92	7,513	69.62	5	0	0	0.0–43.45

Total	2,114	1,584,607	30,125.69	1,185	422	35.6	32.94–38.38

Of the 197 individuals exposed to SARS-CoV-2, only 23 had undergone a PCR test, with 11 of them testing positive, resulting in a positivity rate of 47.8%. Additionally, among these 197 individuals, 98 individuals tested positive on the rapid serological test, representing 49.7% (Table 4).

**TABLE 4:** Factors associated with a positive result on rapid SARS-CoV-2 screening test in univariate and multivariate analysis

Variable	SARS-CoV-2 antibody result				Value from^*a*^:					
	Positive (*n* = 422)		Negative (*n* = 763)		Univariate analysis			Multivariate analysis		
	No.	%	No.	%	OR	95% CI	*P* value	aOR	95% CI	*P* value
Sex										
Female	234	39.1	364	60.9	1.36	1.07–1.73	0.01	1.30	1.01–1.68	0.04
Male	188	32.0	399	68.0	Ref			Ref		
Age (yrs)										
<10	30	34.9	56	65.1	Ref			Ref		
10–19	60	27.6	157	72.4	0.71	0.42–1.22	0.21	0.75	0.43–1.30	0.30
20–29	40	33.6	79	66.4	0.94	0.53–1.69	0.85	0.76	0.41–1.39	0.37
30–39	62	38.3	100	61.7	1.16	0.67–2.00	0.56	1.06	0.60–1.87	0.85
40–49	72	38.1	117	61.9	1.15	0.67–1.95	0.61	0.90	0.52–1.58	0.72
50–59	70	38.3	113	61.7	1.16	0.68–1.97	0.59	1.00	0.57–1.75	1.00
60–69	51	39.5	78	60.5	1.22	0.69–2.15	0.49	1.01	0.55–1.87	0.97
70–79	26	40.0	39	60.0	1.24	0.64–2.42	0.52	0.99	0.48–2.04	0.98
≥80	11	31.4	24	68.6	0.86	0.37–1.98	0.72	0.69	0.28–1.68	0.41
Exposure to SARS-CoV-2 in 2020										
Yes	98	49.7	99	50.3	2.03	1.49–2.76	<0.001	1.34	0.95–1.90	0.09
No	324	32.8	664	67.2	Ref			Ref		
COVID-19 symptoms in 2020										
Yes	207	55.1	169	44.9	3.38	2.62–4.37	<0.001	2.9	2.22–3.80	<0.001
No	215	26.6	594	73.4	Ref			Ref		
Comorbidities										
Yes	111	44.2	140	55.8	1.59	1.20–2.11	0.01	1.32	0.94–1.85	0.10
No	311	33.3	623	66.7	Ref			Ref		
COVID-19 screening and/or diagnostic test in 2020										
Yes	61	57.5	45	42.5	2.7	1.80–4.04	<0.001	1.83	1.18–2.85	0.008
No	361	33.5	718	66.5	Ref			Ref		
PCR										
Positive	15	93.8	1	6.2	12.5	1.4–111.84	0.012^*b*^			
Negative	12	54.5	10	45.5	Ref					
Serology										
Positive	12	66.7	6	33.3	2.84	0.91–8.9	0.10^*b*^			
Negative	19	41.3	27	58.7	Ref					
CT scan										
Positive	13	92.9	1	7.1	13.0	0.98–172.9	0.06^*b*^			
Negative	3	50.0	3	50.0	Ref					
Antigen test										
Positive	1	100.0	0	0.0			0.29^*b*^			
Negative	1	16.7	5	83.3						

a OR, crude odds ratio; aOR, adjusted odds ratio; Ref, reference category.

b Fisher test.

The rapid diagnostic test was also positive in 207 of 376 individuals (55.1%) who reported having symptoms suggestive of COVID-19 in the previous year. Finally, only 38 people (3.2%) underwent a PCR test, 16 of whom tested positive, with 15 of them also testing positive on the rapid diagnostic test, resulting in a concordance rate of 93.8% (Table 4).

### Factors associated with seropositivity.

The results of the univariate analysis revealed several factors that were significantly associated with a positive result on the rapid SARS-CoV-2 screening test. These factors included being female (odds ratio [OR] = 1.36; 95% CI, 1.07 to 1.73; *P* = 0.01), previous exposure to SARS-CoV-2 (OR = 2.03; 95% CI, 1.49 to 2.76; *P* < 0.001), presence of COVID-19 symptoms (OR = 3.38; 95% CI, 2.62 to 4.37; *P* < 0.001), presence of comorbidities (OR = 1.59; 95% CI, 1.20 to 2.11; *P* = 0.01), undergoing COVID-19 screening and/or diagnostic testing (OR = 2.7; 95% CI, 1.80 to 4.04; *P* < 0.001), and a history of positive PCR (OR = 12.5; 95% CI, 1.4 to 111.84; *P* = 0.012) (Table 4). In multivariate analysis, female gender (adjusted odds ratio [aOR] = 1.30; 95% CI, 1.01 to 1.68; *P* = 0.04), presence of COVID-19 symptoms (aOR = 2.9; 95% CI, 2.22 to 3.80; *P* < 0.001), and undergoing COVID-19 screening and/or diagnostic test (aOR = 1.83; 95% CI, 1.18 to 2.85; *P* = 0.008) were significant predictors of a positive rapid SARS-CoV-2 screening test (Table 4).

## DISCUSSION

In our study, the seroprevalence rate of COVID-19 in Oran Province was 35.6% (95% CI, 32.9 to 38.4). Thus, taking into account the population of Oran, the estimated number of COVID-19 cases in the province would be 564,121 cases, which is 44.5 times higher than the number of PCR-confirmed cases, which was 12,665 cases as of 20 January 2021, according to the Health and Population Directorate of the province (7).

Regarding the COVID-19 pandemic worldwide, a literature review (6) conducted between 1 January and 12 August 2020 showed that the proportion of COVID-19 cases detected by seroprevalence surveys was significantly higher than the proportion of cases diagnosed by PCR. Thus, the estimated seroprevalence was 0.56 to 717 times higher than the cumulative number of reported cases. In half of the studies, the prevalence was 10 times higher (6).

For example, in Iran, a study conducted in the province of Guilan (8) from 11 to 19 April 2020 found an adjusted prevalence rate of 22.2% (95% CI, 16.4 to 28.5). This rate was 2.8%, nearly 10 times lower, in a study conducted in Santa Clara County, USA, from 3 to 4 April 2020, with an estimated number of cases 50 to 85 times higher than the number of cases confirmed by PCR (4). It should be noted that this study has received several criticisms related to the small sample size and low statistical power (9).

Furthermore, a study conducted in the city of Kobe, Japan, from 31 March to 7 April 2020 found an age and sex-adjusted seroprevalence rate of 2.7% (95% CI, 1.8 to 3.9), which is 396 to 858 times higher than the number of PCR-confirmed COVID-19 cases in the same city (10).

The situation in Italy was not far from the rates recorded in Japan. Indeed, a study conducted from 24 February to 8 April 2020 among blood donors showed a clear increase in the adjusted seroprevalence, which increased from 2.7% (95% CI, 0.3 to 6.0) to 5.2% (95% CI, 2.4 to 9.0) (11).

It should be noted, however, that few seroprevalence studies had been conducted in Africa, according to Rostami’s literature review (12). The review showed that seroprevalence varied from 4.62% (95% CI, 1.71 to 9.78) in Libya to 5.62% (95% CI, 4.83 to 6.49) in Kenya (12).

In contrast, a study conducted in the Democratic Republic of Congo from May to August 2020 found an estimated anti-SARS-CoV-2 seroprevalence of 40.8% among travelers and workers requiring a medical certificate, which represents a high rate compared to the previously cited studies (13), Several factors can account for variations in COVID-19 seroprevalence rates observed across different countries.

In the Middle East and North Africa (MENA) region, discrepancies in COVID-19 seroprevalence rates between studies can be attributed to variations in testing protocols and methods. A recent population-based study conducted in Tunisia between March and April 2021 found a seroprevalence rate of 38.0%, which is similar to the rate observed in our study, where two enzyme-linked immunosorbent assays (ELISAs) developed at the Pasteur Institute of Tunis were used to detect SARS-CoV-2 antibodies (14).

Similarly, a cross-sectional, community-based study conducted in November and December 2020 in Aden, located in southern Yemen, using Healgen COVID-19 rapid diagnostic test cassettes and ELISA for confirmation, revealed a COVID-19 prevalence rate of 27.4% (15).

Furthermore, the duration of a study can also affect seroprevalence rates, as seen in the El-Ghitani study conducted in Egypt over 6 months between January and June 2021, which coincided with the second and third waves of the COVID-19 pandemic and reported a seroprevalence rate of 53.6% (16).

Moreover, the evolution of the epidemic over time can explain differences in seroprevalence rates. For example, a study in Jordan by Bellizzi et al. (17) showed that the seroprevalence rate increased from 0.3% in August to 7% in October and reached 34.2% by the end of 2020.

Finally, variations in seroprevalence rates can also be attributed to the sampling strategy. A study conducted in Riyadh, Saudi Arabia, in June 2020 reported a seroprevalence rate of 7.8% for COVID-19, but the study sample only included hospitalized patients and healthy blood donors and did not represent the general population (18).

In our study, eight municipalities had recorded seroprevalence rates higher than 73%. However, the study of the relationship between population density and COVID-19 seroprevalence revealed a highly significant positive linear correlation between these two variables (*P* < 0.001). This strong correlation indicates that population density has a significant influence on the number of positive rapid COVID-19 tests and suggests that densely populated areas may be more susceptible to the risk of disease spread. The study conducted by Wong and Li (19) confirms this finding, showing that population density at the county level is a reliable predictor of total infection cases.

### Factors associated with SARS-CoV-2 seropositivity.

In our study, factors associated with SARS-CoV-2 seropositivity in multivariate analysis were female gender, presence of COVID-19 symptoms, and undergoing COVID-19 screening and/or diagnostic test in 2020.

Several studies have identified multiple factors associated with seropositivity, such as sex (20, 21), age (20, 21), obesity (20), exposure to more than one case among nonhousehold contacts (22), presence of multiple comorbidities (20), and previous performance of a PCR test (23).

Thus, the results of our study suggest that anti-SARS-CoV-2 seroprevalence is important compared to the incidence figures of the disease in Oran. Therefore, conducting other prevalence surveys allows tracking the evolution of seroprevalence over time and detecting the establishment of herd immunity, which, once achieved, can indirectly protect vulnerable individuals (24). However, if immunity is distributed heterogeneously within the population, clusters may appear among certain vulnerable individuals (24). Thus, the vaccination strategy could be directed toward the least immunized areas.

### Strengths of the study.

It should be noted that prevalence surveys provide a better estimate of the extent of an epidemic than a surveillance system based on cumulative incidence (4).

In our study, the sample is representative of the population of Oran, given that the cross-sectional survey was based on a four-stage cluster random sampling, which eliminates any selection bias.

In our study, antibody detection was performed using the prick test, similar to some countries, such as the United States (4), that have used this test widely in SARS-CoV-2 screening surveys in the general population. Prick tests are easier to perform and less costly than serological tests (ELISAs), which require taking blood samples and transporting them to the laboratory for analysis, representing a major drawback in terms of feasibility, acceptability, and time (25).

### Weaknesses of the study.

One potential weakness of our study is that we relied on measuring the presence of antibodies in the blood as a marker of immunity to the infection. As it is known that the level of IgG reaches its peak between the 4th and 6th month after infection (26, 27) and then gradually decreases over time (24), it is possible that our study did not capture the full extent of immunity in our study participants. Additionally, the rate of decrease in antibody levels over time may vary depending on individual factors like age and overall health, which could have impacted our results. Therefore, our study may have underestimated the true level of immunity to the infection in our study population.

In some studies, household sampling surveys as a sampling unit may lead to an overestimation of COVID-19 prevalence, as one infected person in the household can transmit the virus to all family members (8).

On the other hand, recruiting nonhospitalized patients, the majority of whom were asymptomatic at the time of the survey, could result in low or undetectable antibody titers, especially if the time elapsed between infection and testing is long (28). Additionally, the accuracy and reliability of rapid serological tests may vary depending on the type of test and other factors.

Ultimately, it should be noted that the results of our study are only representative of Oran Province and cannot be generalized to the rest of the country. Therefore, it is important to conduct a national prevalence survey to estimate the true extent of the COVID-19 epidemic in Algeria.

Finally, our study indicates that the actual number of SARS-CoV-2 infections in Oran is significantly higher than the number of PCR-confirmed cases, which only represents the tip of the iceberg. Moreover, the study shows that prevalence varies between different municipalities, suggesting that specific preventive strategies should be adopted according to the regions. Therefore, to achieve optimal herd immunity against COVID-19, it is advisable to target regions with lower disease prevalence for mass vaccination campaigns. Vaccination against COVID-19 remains the best way to protect against the disease, as the available vaccines are effective in preventing severe forms of the illness, hospitalizations, and COVID-19-related deaths. Further seroprevalence studies in other regions of the country would provide a more comprehensive estimate of the problem and help tailor the response to the epidemic at the national level.

## MATERIALS AND METHODS

### Study type, location, and period.

This was a cross-sectional study of anti-SARS-CoV-2 seroprevalence that included all 26 municipalities of Oran Province. The study specifically targeted household members and was conducted from 7 January to 20 January 2021, during the second wave of the epidemic when the Alpha variant was circulating in Algeria. It was carried out prior to the start of the national anti-COVID-19 vaccination campaign, which began on 30 January 2021 (1).

The Orient Gene kit was used to detect the presence of antibodies directed against the spike protein of the SARS-CoV-2 virus. These antibodies can be detected in the blood of people who have been infected with the virus and have developed an immune response.

This test was chosen due to its ease of use and availability at the health department of our province.

### Eligibility criteria.

Subjects participating in the study were selected using a four-stage cluster random sampling technique after random selection in each municipality of districts, neighborhoods, households, and individuals within each household. Stratification by age and sex categories was performed to increase the chances of obtaining a representative sample of the population of Oran Province. The selected household members were invited to undergo a blood test using a rapid COVID-19 IgG/IgM test cassette from Orient Gene Biotech (sensitivity, 90.5%; specificity, 100.0%) (29) and to complete a preestablished standardized questionnaire.

### Ethical aspects.

The study was conducted after approval from the Health Directorate of Oran Province. The collection of information and screening tests were performed after obtaining informed consent from the subjects included in the study in accordance with the principles of the Helsinki Declaration with regard to confidentiality of personal information, anonymity, and protection of privacy.

### Technique for conducting a screening test.

The COVID-19 IgG/IgM cassette (whole blood/serum/plasma) is a lateral flow immunochromatographic test. The test uses a human anti-IgM antibody, anti-human IgG, and rabbit IgG, mobilized on a nitrocellulose strip. The technique consists of taking a sample of whole blood, transferring the collected whole blood to the sample well of the test device, and adding 2 drops of sample buffer to the buffer well. After the appearance of colored lines, the result is read within 10 min.

In order to avoid potential measurement biases, the investigators received a training session on the conduct and interpretation of the screening test just before the start of the survey.

### Study variables.

Before conducting the serological test, the following variables were collected through direct interview: gender, age, address, locality, history of exposure to SARS-CoV-2, type of contact (family, professional, neighborhood, or other), presence or absence of suggestive clinical signs of COVID-19 in the previous year, different clinical signs in favor of possible previous infection (headaches, cough, asthenia, fever, shortness of breath, anosmia, ageusia, nausea, diarrhea, chills), presence of comorbidities (hypertension, diabetes, cardiovascular diseases, and others), and history of screening or diagnostic testing for COVID-19 in the previous year. The result of the rapid serological test of the prevalence survey (positive or negative) was also recorded, and if positive, the type of antibody (IgM or IgG) was further detailed.

### Sample size calculation.

Based on the population of Oran Province, estimated at 1,584,607 inhabitants, the sample size *n* was calculated using the following formula:

$$n=\text{Deff}\times \frac{\mathrm{Np}\left(1-p\right)}{\frac{d^{2}}{1.96^{2}}\left(N-1\right)+p\left(1-p\right)}$$

Thus, assuming an anticipated disease frequency *p* of 50%, a calculation factor Deff of 1, a precision *d* of 3%, and a corresponding alpha error risk of 5% (E = 1.96), the calculated required number of subjects was 1,067. To mitigate the risk of refusal to participate in the study, the research team opted for a larger sample size of 1,200 subjects.

### Statistical analysis.

The overall seroprevalence, as well as specific seroprevalences by commune, were calculated and presented with their 95% confidence intervals (CIs) based on Wilson’s score, provided by the OpenEpi program (30).

Quantitative variables were compared using Student’s *t* test, and the relationship between qualitative variables was examined using the chi-square test in our study. Both univariate and multivariate analyses were conducted for statistical analysis. Univariate analysis involved calculating odds ratios (ORs) to assess the strength of association between each independent variable and the outcome variable. In the multivariate analysis based on binary logistic regression, adjusted ORs (aORs) were calculated while controlling for potential confounding variables. All statistical analyses were performed using SPSS version 20. A *P* value of less than 0.05 was considered statistically significant for a relationship.

### Data availability.

The data for this study can be accessed at the following link: https://zenodo.org/record/7991424.
